# Multiplanar evaluation of radiological findings associated with acetabular dysplasia and investigation of its prevalence in an Asian population: a CT-based study

**DOI:** 10.1186/s12891-017-1426-3

**Published:** 2017-01-31

**Authors:** Tomohiro Mimura, Kanji Mori, Masahiro Kitagawa, Mariko Ueki, Yuki Furuya, Taku Kawasaki, Shinji Imai

**Affiliations:** Department of Orthopedic Surgery, Shiga University of Medical Science, Tsukinowa-cho, Seta, Otsu, Shiga, 520-2192 Japan

**Keywords:** Acetabular dysplasia, Developmental dysplasia of the hip, Prevalence, Computed tomography, Multiple parameters, Multiplanar

## Abstract

**Background:**

Acetabular dysplasia (AD) is a well-known cause of osteoarthritis (OA) of the hip, with its prevalence previously determined on plain radiography. The prevalence of preexisting AD was reported as 7.3% in a patient-based Asian population. Although computed tomography (CT) could evaluate AD in multiple planes, its prevalence using multiplanar CT images has not been reported. We investigated its prevalence with CT on coronal, axial, and sagittal planes and then determined if adding the axial and sagittal planes enhanced the investigation.

**Methods:**

We retrospectively examined 52 consecutive Japanese individuals (mean age 59.4 years) who had undergone CT for conditions unrelated to hip disorders. The inclusion criteria of CT images were (1) reconstructed axial slice thickness of ≤1 mm and (2) normal pelvic rotations and tilt. Exclusion criteria were (1) age <20 years, (2) neither hip center could be clearly detected, (3) evidence of hip OA. The parameters used to define AD on the coronal plane were the center–edge angle, Sharp angle, acetabular index, acetabular depth ratio, and acetabulum head index. The anterior and posterior acetabular sector angles were used as axial parameters and the vertical-center-anterior margin angle as the sagittal parameter. AD prevalence was calculated using multiplanar images and then compared with the previously reported Asian prevalence using 95% confidence intervals (CI). In this study, we defined “prevalence” as the proportion of subjects who had AD in at least one hip.

**Results:**

The mean prevalence of AD on coronal, axial, and sagittal planes was 16.9, 15.4, and 7.7%, respectively. The lowest prevalence found by combining the three planes was 25.0% (95% CI 15.2–38.2%). This prevalence was significantly higher than that in the previously reported Asian population (7.3%).

**Conclusions:**

At the lowest estimate, the prevalence of AD evaluated in three planes was more than twice as high as the previously reported prevalence in Asians when we investigated its prevalence using multiplanar images. The prevalence of AD in the axial and sagittal planes was not negligible. We therefore suggest that it is important to add axial and sagittal planes’ data when investigating the prevalence of AD.

## Background

Acetabular dysplasia (AD) is a well-known cause of osteoarthritis (OA) of the hip [1, 2]. The morphological abnormalities associated with AD result in instability of the hip joint, leading to labral tears, cartilage degeneration, and development of OA. AD is the most common cause of hip OA, especially in Asian countries [3, 4]. The parameters employed for diagnosing AD on the coronal plane are the center–edge (CE) angle [5], Sharp angle [6], acetabular index [7], acetabular depth ratio (ADR) [8], and acetabulum head index (AHI) [9]. The anterior (AASA) and posterior (PASA) acetabular sector angles [10] are used to diagnose AD on the axial plane, and the vertical-center-anterior margin (VCA) angle [11] is used to diagnose AD on the sagittal plane. Although the prevalence of AD has been reported using hip joint radiography, pelvic radiography, or urography, it has been discussed only in terms of the coronal plane. Umer et al. [12] reported that the prevalence of AD was 7.3% (CE angle <20°) using pelvic radiography in a patient-based Asian population.

Computed tomography (CT) and magnetic resonance imaging (MRI) provide images not only of the coronal plane but also the axial and sagittal planes. No reports, however, have shown the prevalence of AD using multiplanar CT or MRI investigations. Because AD is an important etiology of OA of the hip that can reduce a patient’s healthy life-span, we thought that the prevalence of AD should not be discussed based only on coronal plane investigations. We therefore investigated the morphological features of the acetabulum in a convenience sample of Japanese patients using reconstructed, high-resolution CT images in the coronal, axial, and sagittal planes. The aims of this study were to investigate the prevalence of AD on each plane and then using a combination of the three planes. Based on the results, we assessed the usefulness of adding the axial and sagittal plane for investigating the prevalence of AD in a Japanese population.

## Methods

### Patients and parameters

We conducted a study on patients who had undergone CT imaging of the chest, abdomen, and pelvis including the hip joints. The CT scans had been requested by other departments at our institution for conditions unrelated to hip disorders. The inclusion criteria were as follows: (1) the reconstructed axial slice thickness was ≤1 mm and (2) pelvic rotations and tilt were normal (described later in the section describing standardization of CT images). The exclusion criteria were as follows: (1) age under 20 years; (2) the hip center of either hip could not be clearly detected (e.g., a hip with an elliptical femoral head; and (3) evidence of hip OA in either hip (e.g., the presence of joint space narrowing, osteophytes, or subchondral bone changes, including cysts and sclerosis) [13].

**Fig. 1:**
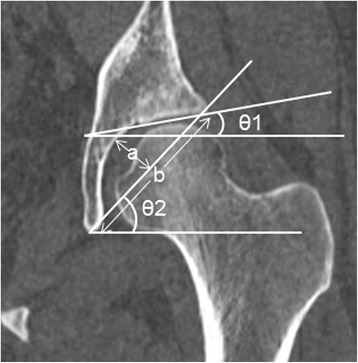
Measurement of the parameters used to define acetabular dysplasia. θ1 is the acetabular index. θ2 is the Sharp angle. *a*/*b* is the acetabular depth ratio (ADR). These parameters were measured on a slice of the hip center in the coronal plane, orthogonal to the standard axial plane. The acetabular index was measured as the angle between the line joining the medial and lateral aspects of the weight-bearing zone and the line parallel to the transverse axis of the pelvis. Sharp angle was measured as the angle between the line joining the lateral aspect of the weight-bearing zone and the inferior point of teardrop and the line parallel to the transverse axis of the pelvis. ADR was calculated by dividing the depth of the acetabulum by the length between the inferior teardrop point and the lateral weight-bearing zone in the coronal plane of the femoral head center, then multiplying by 1000

**Fig. 2:**
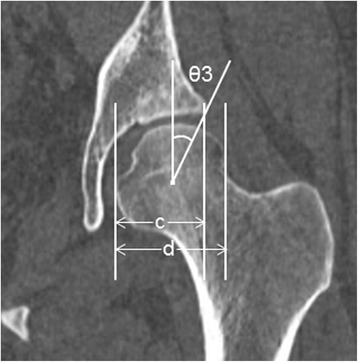
Measurement of the parameters used to define acetabular dysplasia. θ3 is the center–edge (CE) angle. c/d is the acetabulum head index (AHI). These parameters were measured on a slice of the hip center in the coronal plane. CE angle was measured as the angle between the line joining the lateral aspect of the weight-bearing zone and the femoral head center and the line perpendicular to the transverse axis of the pelvis. AHI was calculated by dividing the length from the medial margin of the femoral head to the lateral side of the weight-bearing zone by the femoral head width, then multiplying by 100

**Fig. 3:**
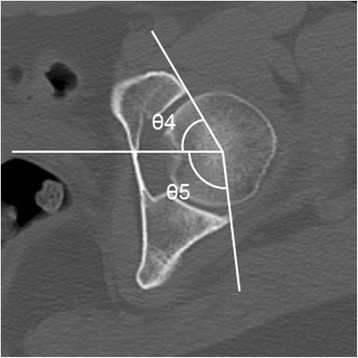
Measurement of the parameters used to define acetabular dysplasia. θ4 and θ5 are the anterior acetabular sector angle (AASA) and posterior acetabular sector angle (PASA), respectively. These parameters were measured on a slice of the hip center in the axial plane. AASA was measured as the angle between the horizontal line joining the bilateral femoral head center and the line joining the anterior margin of the acetabulum and the femoral head center. PASA was measured as the angle between the horizontal line combing the bilateral femoral head center, and the line joining the posterior margin of the acetabulum and femoral head center

**Fig. 4:**
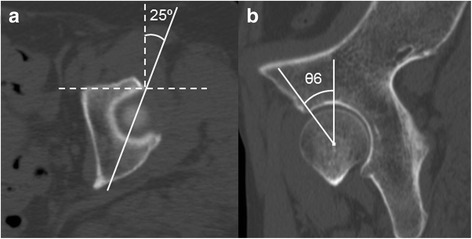
Measurement of the vertical-center-anterior margin (VCA) angle on the sagittal plane. The VCA angle is measured according to the method of Needell et al. [14]. **a** Reference standard axial plane for measuring the VCA angle. *Solid line* is drawn 25° obliquely from the most anterior lateral margin of the acetabulum, which is identified on the axial plane at the opening of the acetabulum. **b** Reconstructed 25° oblique sagittal plane (*solid line* in **a**). θ6 is the VCA angle, which is measured between the line joining the anterior aspect of the weight-bearing zone and the hip center and the perpendicular line from the femoral head center

We retrospectively examined 52 consecutive Japanese patients (29 men, 23 women) who met the above criteria from July 1, 2013 to July 31, 2013. Both hips were analyzed in each patient. We performed detailed analyses of the morphological parameters associated with AD obtained from high-resolution, reconstructed, multi-slice CT images (1 mm thick slices) and then calculated the prevalence of AD. The parameters examined included the CE angle, Sharp angle, acetabular index, ADR, AHI, AASA, PASA, and VCA angle. The CE angle, Sharp angle, acetabular index, ADR, and AHI were measured in the coronal plane of the hip center (Figs. 1 and 2). AASA and PASA were measured on the axial plane of the hip center according to the method described by Anda et al. [10] (Fig. 3). The VCA angle was measured in a 25° oblique sagittal plane of the hip center according to the method described by Needell et al. [14] (Fig. 4). AD was defined as a CE angle <20° [15, 16], Sharp angle >45° [17, 18], acetabular index >14° [19, 20], ADR <250 [15, 17], AHI <75% [15, 21], AASA <50° [22, 23], PASA <90° [22, 23], or VCA angle <20° [13, 16]. We calculated the prevalence of AD by analyzing the anatomical parameters associated with AD in detail using high-resolution, reconstructed, multiplanar CT images. We then compared the prevalence with that of previously reported preexisting AD in an Asian population (7.3%) reported by Umer et al. [12]. The proportion of subjects who had parameters defined as AD in at least one hip was defined as the prevalence.

### Radiological examination and standardization of CT images

All CT images were axial, sequential, and obtained in the supine position without gantry tilt (120 kV, 160 mA, 0.5 s) using a Toshiba Aquilion CX (Toshiba Medical Systems, Tokyo, Japan). The data were reconstructed under conditions suitable for bone evaluation using AquariasNet Viewer software (TeraRecon, San Francisco, CA, USA). This software allowed reconstruction of optimal sagittal, coronal, and axial views as well as three-dimensional reconstructed CT (3D CT) views. We used the 3D CT images to confirm pelvic rotation and tilt. We confirmed (1) the rotation of the coronal plane (to investigate whether the teardrop line was horizontal), (2) the rotation of the axial plane (to examine whether the tip of the coccyx was present above the pubic symphysis) [24], and (3) the neutral pelvic tilt (to investigate the distance between the upper border of the symphysis and the mid-portion of the sacrococcygeal joint, as previously described [24]). In the present study, 32 ± 10 mm in men and 47 ± 10 mm in women were considered neutral.

### Evaluation of the interobserver and intraobserver reliability

The interobserver reliability between the first (TM) and second (TK) observers and intraobserver reliability between the first and second assessments (TM) were evaluated for the first 20 consecutive cases.

### Statistical analysis

All statistical analyses were performed using SPSS Statistics 22.0 for Windows (SPSS, Chicago, IL, USA). The 95% confidence interval (CI) based on the score test for prevalence was estimated. Intraclass correlation coefficients (ICCs) were calculated to evaluate the interobserver and intraobserver reliability. The ICC was interpreted using the categories of agreement suggested by Landis and Koch [25], where ≤0.40 is unacceptable, 0.41–0.60 is moderate, 0.61–0.80 is substantial, and ≥0.80 is almost perfect agreement. The significance of differences between men and women was evaluated using the Mann–Whitney *U*-test and a *χ* ^2^ test. Values of *P* < 0.05 were considered to indicate statistical significance.

## Results

One male hip was excluded because a bone tumor was identified in the metaphyseal portion. As a result, a total of 103 hips (57 in men, 46 in female) were evaluated. Table 1 shows the mean age and all parameters for the subjects. The mean values of AASA and PASA showed significant differences between male and females. The AASA was smaller in women than in men, and PASA was smaller in men than in women.

**Table 1:** Mean age and value of each parameter of all hips and by sex

Age and parameter	All hips (*n* = 103)	Males (*n* = 57)	Females (*n* = 46)	*P* value*
Age (years)	59.4 ± 14.8 (56.5–62.2)	60.3 ± 14.7 (56.4–64.1)	58.2 ± 15.3 (53.8–62.6)	0.604
CE angle (°)	31.1 ± 7.1 (29.7–32.4)	31.6 ± 7.0 (29.9–33.4)	30.2 ± 7.3 (28.1–32.3)	0.367
Sharp angle (°)	40.1 ± 3.9 (39.3–40.8)	39.7 ± 4.2 (38.7–40.7)	40.5 ± 3.7 (39.5–41.6)	0.139
Acetabular index (°)	7.0 ± 9.8 (5.1–8.9)	6.0 ± 6.5 (4.4–7.6)	6.8 ± 6.6 (4.8–8.7)	0.346
ADR	308.3 ± 47.9 (298.7–317.2)	305.6 ± 43.2 (295.0–316.2)	309.3 ± 52.3 (294.2–324.5)	0.637
AHI (%)	81.9 ± 6.0 (80.7–83.2)	81.6 ± 6.1 (80.1–83.1)	82.4 ± 6.3 (80.5–84.2)	0.673
AASA (°)	59.3 ± 7.9 (57.7–60.8)	60.8 ± 6.8 (59.1–62.5)	57.3 ± 8.9 (54.8–59.9)	0.025
PASA (°)	98.6 ± 9.5 (96.8–100.5)	96.7 ± 10.2 (94.2–99.2)	100.9 ± 8.2 (98.5–103.3)	0.043
VCA angle (°)	31.1 ± 6.0 (29.9–32.2)	31.2 ± 5.5 (29.8–32.5)	30.9 ± 6.7 (29.0–32.9)	0.831

Data are shown means ± SD with 95% confidence interval in parenthesis. *AD* acetabular dysplasia, *CT* computed tomography, *CE* center edge, *ADR* acetabular depth ratio, *AHI* acetabulum head index, *AASA* anterior acetabular sector angle, *PASA* posterior acetabular sector angle, *VCA* vertical-center-anterior margin angle. *Males *vs* females, evaluated with the Mann–Whitney *U* test. *P* values < 0.05 were considered statistically significant

Table 2 shows the prevalence of AD defined by each parameter for all subjects and by sex. The prevalence of AD defined by the CE angle, Sharp angle, acetabular index, ADR, AASA, and VCA angle was higher in women than in men. Among these parameters, there was a significant difference only for AASA. For the coronal parameters, the prevalence among all subjects was lowest when defined by the CE angle and highest with ADR. The prevalence of AD on the coronal plane was 11.5–23.1% for all subjects, 10.3–17.2% in men, and 13.0–30.4% in women. On the axial plane, the prevalence of AD was 13.5–17.3% for all subjects, 6.9–23.1% in men, and 4.3–30.4% in women. The mean prevalence of AD defined with each parameter was 16.9% (15.1% men, 19.1% women) on the coronal plane, 15.4% (13.8% men, 17.4% women) on the axial plane, and 7.7% (3.4% men, 13.0% women) on the sagittal plane.

**Table 2:** Prevalence of acetabular dysplasia cases, by each parameter in three planes

Definition of AD	All subjects (*n* = 52)	Males (*n* = 29)	Females (*n* = 23)	*P* value*
Coronal plane				
CE angle <20°	11.5% (6/52) (95% CI: 5.4–23.0%)	10.3% (3/29) (95% CI: 3.6–26.4%)	13.0% (3/23) (95% CI: 4.5–32.1%)	0.762
Sharp angle >45°	17.3% (9/52) (95% CI: 9.4–29.7%)	13.8% (4/29) (95% CI: 5.5–26.3%)	21.7% (5/23) (95% CI: 9.7–41.9%)	0.452
Acetabular index >14°	17.3% (9/52) (95% CI: 9.4–29.7%)	17.2% (5/29) (95% CI: 7.6–34.5%)	17.4% (4/23) (95% CI: 7.0–37.1%)	0.989
ADR <250	23.1% (12/52) (95% CI: 13.7–36.1%)	17.2% (5/29) (95% CI: 7.6–34.5%)	30.4% (7/23) (95% CI: 15.6–50.9%)	0.262
AHI <75%	15.4% (8/52) (95% CI: 8.0–27.5%)	17.2% (5/29) (95% CI: 7.6–34.5%)	13.0% (3/23) (95% CI: 4.5–32.1%)	0.677
Axial plane				
AASA <50°	17.3% (9/52) (95% CI: 9.4–29.7%)	6.9% (2/29) (95% CI: 1.9–22.0%)	30.4% (7/23) (95% CI: 15.6–50.9%)	0.026
PASA <90°	13.5% (7/52) (95% CI: 6.7–25.3%)	20.7% (6/29) (95% CI: 9.8–38.4%)	4.3% (1/23) (95% CI: 0.8–21.0%)	0.086
Sagittal plane				
VCA angle <20°	7.7% (4/52) (95% CI: 3.0–18.2%)	3.4% (1/29) (95% CI: 0.6–17.2%)	13.0% (3/23) (95% CI: 4.5–32.1%)	0.197

*AD* acetabular dysplasia, *CE* center edge, *ADR* acetabular depth ratio, *AHI* acetabulum head index, *AASA* anterior acetabular sector angle, *PASA* posterior acetabular sector angle, *VCA* vertical-center-anterior margin angle. *Male *vs* female subjects, evaluated with a *χ* ^2^ test. *P* values < 0.05 were considered statistically significant

Table 3 shows the prevalence of AD with each parameter when calculated from a combination of the planes (coronal, axial, and sagittal). In all subjects, the prevalence was lowest (25.0%) when defined with a CE angle <20°, AASA <50°, or VCA angle <20° and highest (36.5%) when defined by ADR <250, PASA <50°, or VCA angle <20° in all three planes. In men, the prevalence was lowest (13.8%) when defined by CE angle <20°, AASA <50°, or VCA angle <20° and highest (34.5%) when defined by ADR <250, PASA <90°, or VCA angle <20° in all three planes. In women, the prevalence was lowest (26.1%) when defined by a CE angle <20°, PASA <90°, or VCA angle <20° and highest (47.8%) when defined by ADR <250, AASA <50°, or VCA angle <20° in all three planes. The prevalence defined by a combination of parameters on three planes was higher in women than in men, except for a CE angle <20°, PASA <50°, or VCA angle <20°. There were significant differences between men and women for three combinations: “CE angle <20°, AASA <50°, or VCA angle <20°”; “Sharp angle <20°, AASA <50°, or VCA angle <20°”; “ADR <250, AASA <50°, or VCA angle <20°.”

**Table 3:** Prevalence of AD calculated from all combinations with each parameter in the three planes (coronal, axial, sagittal)

Definition of AD	All subjects (*n* = 52)	Males (*n* = 29)	Females (*n* = 23)	*P* value*
CE angle <20°, AASA <50°, or VCA angle <20°	25.0% (13/52) (95% CI: 15.2–38.2%)	13.8% (4/29) (95% CI: 5.5–30.6%)	39.1% (9/23) (95% CI: 22.2–59.2%)	0.036
CE angle <20°, PASA <90°, or VCA angle <20°	26.9% (14/52) (95% CI: 16.8–40.3%)	27.6% (8/29) (95% CI: 14.7–45.7%)	26.1% (6/23) (95% CI: 12.5–46.5%)	0.903
Sharp angle >45°, AASA <50°, or VCA angle <20°	28.8% (15/52) (95% CI: 18.3–42.3%)	17.2% (5/29) (95% CI: 7.6–34.5%)	43.5% (10/23) (95% CI: 25.6–63.2%)	0.038
Sharp angle >45°, PASA <90°, or VCA angle <20°	30.8% (16/52) (95% CI: 19.9–44.3%)	27.6% (8/29) (95% CI: 14.7–45.7%)	34.8% (8/23) (95% CI: 18.8–55.1%)	0.576
Acetabula index >14°, AASA <50°, or VCA angle <20°	28.8% (15/52) (95% CI: 18.3–42.3%)	20.7% (6/29) (95% CI: 9.8–38.4%)	39.1% (9/23) (95% CI: 22.2–59.2%)	0.144
Acetabula index >14°, PASA <90°, or VCA angle <20°	28.8% (15/52) (95% CI: 18.3–42.3%)	27.6%(8/29) (95% CI: 14.7–45.7%)	30.4% (7/23) (95% CI: 15.6–50.9%)	0.821
ADR <250, AASA <50°, or VCA angle <20°	32.7% (17/52) (95% CI: 21.5–46.2%)	20.7% (6/29) (95% CI: 9.8–38.4%)	47.8% (11/23) (95% CI: 29.2–67.0%)	0.038
ADR <250, PASA <90°, or VCA angle <20°	36.5% (19/52) (95% CI: 24.8–50.1%)	34.5% (10/29) (95% CI: 19.9–52.7%)	39.1% (9/23) (95% CI: 22.2–59.2%)	0.729
AHI <75%, AASA <50°, or VCA angle <20°	23.1% (15/52) (95% CI: 18.3–42.3%)	20.7% (6/29) (95% CI: 9.8–38.4%)	39.1% (9/23) (95% CI: 22.2–59.2%)	0.144
AHI <75%, PASA <90°, or VCA angle <20°	28.8% (15/52) (95% CI: 18.3–42.3%)	27.6% (8/29) (95% CI: 14.7–45.7%)	30.4% (7/23) (95% CI: 15.6–50.9%)	0.821

*AD* acetabular dysplasia, *CE* center edge, *AASA* anterior acetabular sector angle, *VCA* vertical-center-anterior margin angle, *PASA* posterior acetabular sector angle, *ADR* acetabular depth ratio, *AHI* acetabulum head index. *Male *vs* female subjects, evaluated with a *χ* ^2^ test. *P* values < 0.05 were considered statistically significant

The ICC values for intraobserver reliability were as follows: CE, 0.91; Sharp angle, 0.94; acetabular index, 0.88; ADR, 0.89; AHI, 0.79; AASA, 0.77; PASA, 0.85; VCA angle, 0.61. The ICC values for the interobserver reliability also were as follows: CE, 0.83; Sharp angle, 0.94; acetabular index, 0.91; ADR, 0.93; AHI, 0.69; AASA, 0.80; PASA, 0.92; VCA angle, 0.64.

## Discussion

AD is one of the etiologies of hip OA, which is a major disease that affects the healthy life-span of a population [3, 26–28]. Previous reports, however, have based the prevalence of AD only on data derived from plain radiography. We conducted a detailed evaluation of the prevalence of AD using multiplanar CT images. To the best of our knowledge, this is the first study to discuss the prevalence of AD using the coronal, axial, and sagittal planes in combination. In this study, “prevalence” was defined as the proportion of subjects who had AD in at least one hip, which distinguishes it from the definition stating it is the proportion of hips “among all of the hips”.

We found few studies that reported AD prevalence as just defined in an Asian population. Umer et al. [12] reported the prevalence of AD in a Singaporean population. Their report was a patient-based study with subjects similar to those in our study. They evaluated 261 asymptomatic patients (mean age 60 years, range 16–99 years), most of whom were trauma patients. They excluded patients who presented with hip pain. Pelvic radiography and a CE angle <20° was employed for their definition of AD. They reported that the prevalence of AD was 7.3% (19/261 patients).

In the present study, we found that the prevalence, as defined with the CE angle alone, was 11.5% (95% CI 5.4–23.0%). According to this result, our prevalence calculated from the CE angle was similar to the 7.3% (preexisting AD prevalence in Asians) reported by Umer et al. [12]. However, the prevalence of AD defined with AASA <50° on the axial plane was 17.3% (95% CI 9.4–29.7%). This lower limit of 95% CI was higher than 7.3%. The prevalence on the sagittal plane, however, was 7.7%. All AD prevalence data defined using a combination of the three planes were much higher than that reported by Umer et al. (Table 3). The lowest prevalence defined using each parameter in the three planes was 25.0% (95% CI 15.2–38.2%). Taking this lower limit of 95% CI into consideration, the prevalence of AD evaluated using data from three planes was at least twice as high as the previous prevalence (7.3%) in Asians. Therefore, we believe that we should pay attention to the prevalence of AD on the axial and sagittal planes as well as the coronal plane.

Two large studies from Western countries reported the prevalence of AD in population based-studies using pelvic radiography [17, 18]. Jacobsen et al. [17] studied the prevalence of AD in a normal Danish population. They found an AD prevalence of 3.4% with CE ≤20°, 6.4% with Sharp angle ≥45°, 3.0% with AHI <75%, and 8.8% with ADR ≤250 in 3859 subjects. Engesaeter et al. [18] reported the prevalence of AD in a normal Norwegian population. They surveyed 2027 young adults and found that the prevalence of AD was 3.3% with CE <20°, 13.0% with Sharp angle >45°, 5.8% with AHI <75%, and 12% with ADR ≤250. We recognized that it is difficult to compare our results with these results directly because our prevalence was calculated from a patient-based population and theirs were calculated from a normal population. It is in line, however, with our results that the prevalence of AD defined using the CE angle is low and the prevalence defined using ADR is high. We believe that defining the prevalence of AD based only the CE angle is not accurate and has a risk of underestimating the prevalence.

AD was generally seen more often in women [17, 26]. In the present study, the prevalence among women was significantly higher than that of men when using only AASA as a parameter for defining AD (Table 2). There were no other significant differences in the mean values of the parameters, except AASA and PASA, between men and women (Table 1). We think that these findings with respect to axial parameters maybe be related to a retroverted acetabulum (i.e., a pincer deformity of femoroacetabular impingement). Considering these different AASA and PASA results in men and women, in our study the male acetabulum was significantly more retroverted than that in women. These results are in line with those in previous reports, which showed that a retroverted acetabulum was detected in men significantly more often than in women [29, 30]. In other words, it is suggested that there is posterior undercoverage of the acetabulum in men and anterior undercoverage in women. We suggest that the morphological undercoverage of the acetabulum on the axial plane is a significant, clinically important finding when discussing AD. At this point, we must emphasize that these parameters cannot be evaluated using plain radiography. Additionally, we found that three combinations—CE angle <20°/AASA <50°/VCA angle <20°; Sharp angle <20°/AASA <50°/VCA angle <20°; ADR <250/AASA <50°/VCA angle <20°—showed a significantly higher AD prevalence in women than in men (Table 3). We suggest that these findings were also affected differently by axial plane parameters in men and women.

We report herein the prevalence of AD based on CT measurements. We recognized that there was the discrepancy in the values found by CT and plain radiography. For example, two methods for measuring the CE angle on plain radiography—the classic Wiberg CE angle [5] and Ogata’s CE angle [31]—have been reported. Chadayammuri et al. [32] reported a discrepancy in the values of the CE angle between plain radiography and CT. They reported that the CE angle measured with CT imaging was 2.1° larger than that found using Ogata’s CE angle on radiography. In addition, Omeroglu et al. [33] reported a discrepancy in the CE angle between Wiberg’s CE angle and Ogata’s CE angle determined on plain radiography. They reported that the measured values of Wiberg’s CE angle were 8.9° larger than those determined using Ogata’s CE angle. These discrepancies were due to a difference in the measuring point on the lateral edge of the acetabulum. The measuring point of Ogata’s CE angle is the lateral weight-bearing sclerotic zone (sourcil) of the acetabulum on radiography, whereas the measuring point of the classic Wiberg’s CE angle is the most lateral rim of the acetabulum on radiography. Considering these facts, our results using the CE angle, Sharp angle, acetabular index, and AHI might slightly overestimate the prevalence of AD based on the studies that measured these parameters using Wiberg’s lateral point. However, we believe that CT imaging is more suitable than plain radiography from the viewpoint of correctly measuring the parameters to evaluate AD because we can easily detect the bony morphological features of the acetabulum, especially the lateral point of the acetabular dome on CT images. We can also evaluate the correct acetabular bony coverage in the center of the hip joint. Finally, the ICC values for the interobserver reliabilities of the CE angle and acetabular index in the present study were 0.83 and 0.91, respectively. Mineta et al. [34] reported that the corresponding values for CT examination were 0.94 and 0.97, respectively. Mast et al. [35] and Tan et al. [36] reported that the ICC values for the interobserver reliability of CE angle evaluated in plain radiography were 0.73 and 0.51, respectively. Mast et al. [35] and Tannast et al. [37] reported that the ICC values for the interobserver reliability of acetabular index evaluated on plain radiography ware 0.45 and 0.61, respectively. We think that CT imaging is more suitable for measuring the morphological parameters needed to evaluate AD—that CT images provide a more correct prevalence of AD than plain radiography.

This study has some limitations. First, this study was not population-based. It was patient-based. We investigated the CT data of patients (e.g., digestive, circulatory, urological, gynecological, hematological, respiratory, kidney disease), not those of a general population. Therefore, our cohort was a convenience sample. This is an inescapable limitation of this study. We understood that it would have been better to employ healthy volunteers. It would be unethical, however, to ask volunteers to undergo CT examination only to provide data for this study. We believe that exclusion of patients of hip disorders and/or hip OA allowed us to limit this potential limitation as much as possible. In fact, our prevalence of AD defined as CE <20° was low on the coronal plane, and our prevalence defined as ADR <250 was high. These results are in line with those of population-based studies of Western populations.

Second, the investigation period was short, the sample size relatively small, and the study was performed in a single hospital. Therefore, we expect that in the future similar, multicenter studies with larger sample sizes will be reported.

Third, the ICC values for intraobserver and interobserver reliability regarding the VCA angle were relatively lower than for the other parameters. We thought that the reason was the method of measurement for the VCA angle, which was clearly more complex than those for the other parameters (Fig. 4). Finally, the number of parameters employed for the definition of AD was different in each plane. Five parameters were employed on the coronal plane, two on the axial plane, but only one on the sagittal plane.

Stubbs et al. [38] reported that reconstructed CT images provide a better screening tool for identifying AD than traditional radiographic assessment. We found that evaluating AD in the axial and sagittal planes was useful, and the results were not negligible (Tables 2 and 3). As a result, we found that the prevalence of AD was higher than the preexisting AD prevalence based on plain radiography. We emphasize that multiplanar evaluation is useful for understanding the morphological features of AD and is needed to investigate the prevalence of AD in detail. Important measures should be taken to prevent, or at least retard, the onset of OA, thereby avoiding progression to symptomatic hip OA, which could affect patients’ quality of life and be a considerable health care burden.

## Conclusions

We investigated the prevalence of AD using multiplanar CT images in a Japanese population, which showed that the prevalence of AD on coronal, axial, and sagittal plane was 16.9, 15.4, and 7.7%, respectively. Even at the lowest estimate, the prevalence evaluated when combing the data for all three planes was more than twice as high as the preexisting AD prevalence in an Asian population using only the coronal plane. We suggest that the prevalence of AD in the axial and sagittal planes is not negligible. Hence, it is important to add axial and sagittal plane data when investigating the prevalence of AD.

## Abbreviations

AASA: Anterior acetabular sector angle
AD: Acetabular dysplasia
ADR: Acetabular depth ratio
AHI: Acetabulum head index
CE: Center–edge
CI: Confidence interval
CT: Computed tomography
3D CT: Three-dimensional reconstructed CT
ICC: Intraclass correlation coefficient
OA: Osteoarthritis
PASA: Posterior acetabular sector angle
VCA: Vertical-center-anterior margin
